# Influenza in long‐term care facilities

**DOI:** 10.1111/irv.12464

**Published:** 2017-07-26

**Authors:** Louise E. Lansbury, Caroline S. Brown, Jonathan S. Nguyen‐Van‐Tam

**Affiliations:** ^1^ Health Protection and Influenza Research Group Division of Epidemiology and Public Health City Hospital University of Nottingham Nottingham UK; ^2^ Influenza & Other Respiratory Pathogens Programme Division of Communicable Diseases and Health Security WHO Regional Office for Europe UN City Copenhagen Denmark

**Keywords:** antivirals, infection control, influenza, long‐term care, vaccines

## Abstract

Long‐term care facility environments and the vulnerability of their residents provide a setting conducive to the rapid spread of influenza virus and other respiratory pathogens. Infections may be introduced by staff, visitors or new or transferred residents, and outbreaks of influenza in such settings can have devastating consequences for individuals, as well as placing extra strain on health services. As the population ages over the coming decades, increased provision of such facilities seems likely. The need for robust infection prevention and control practices will therefore remain of paramount importance if the impact of outbreaks is to be minimised. In this review, we discuss the nature of the problem of influenza in long‐term care facilities, and approaches to preventive and control measures, including vaccination of residents and staff, and the use of antiviral drugs for treatment and prophylaxis, based on currently available evidence.

## 1 INTRODUCTION

The term “long‐term care facility” (LTCF) encompasses a diverse range of healthcare settings including nursing homes, rehabilitation centres, long‐term care hospitals, psychiatric care facilities and facilities for people with intellectual disabilities.^1^ Although people of all ages may reside in these facilities, the majority of residents are elderly. With the population in Europe aged 85 years and above projected to rise from 14 million currently to 19 million by 2020 and to 40 million by 2050, and the expectation that more than 30% of European citizens will be aged over 60 years by 2050, the proportion of the population in countries at all levels of development which requires long‐term care is only set to increase dramatically over the coming decades.^2^

Outbreaks of seasonal influenza in LTCFs are well recognised, as are the challenges of preventing and controlling influenza outbreaks in these settings. The development of universally applicable guidance on the prevention and control of influenza and other respiratory viruses in LTCFs is difficult due to the huge variation in the size of facilities, patient characteristics, the intensity of care provided and resources available. Although some countries have produced guidance on IPC for use specifically in LTCFs,^3^, ^4^, ^5^, ^6^, ^7^, ^8^, ^9^, ^10^, ^11^ most have not. To help fill this gap, the WHO has recently published a best practice guidance document to support managers of LTCFs in the 53 WHO European Region Member States and which can be tailored according to national and local circumstances^12^ ( http://www.euro.who.int/__data/assets/pdf_file/0015/330225/LTCF-best-practice-guidance.pdf?ua=1).

In this review, we examine the impact of seasonal influenza in LTCFs, and approaches to the prevention and control of outbreaks, as outlined by the advice and evidence we provided in the WHO best practice document.

## 2 THE IMPACT OF INFLUENZA

Persons residing in LTCFs present a population very susceptible to the acquisition and spread of infectious diseases and for whom the consequences of infection may be serious. Nursing home residents are at greatest risk due to their overall frailty, close quarter living arrangements, shared caregivers, and opportunities for introduction of healthcare‐associated infections and the spread of pathogens to other facilities through resident transfers and the movement of staff and visitors in and out of the home.^13^, ^14^

Outbreaks of influenza caused by both influenza A and B viruses are well documented in LTCFs, and may be explosive,^15^ with high mortality, highlighting the need for early recognition and prompt initiation of control measures. Accurate measurement of the burden of influenza is heavily influenced by circulating types and subtypes of virus and may vary between communities and between institutions so studies that attempt to estimate this burden require temporal, geographical and institutional breadth.^16^ Older studies, relying on culture‐based detection techniques, may have underestimated total burden. A review of 206 published infectious outbreaks in elderly care facilities across 19 countries over 40 years identified 37 different pathogens, but influenza viruses caused the largest number of outbreaks (23%).^17^ In the 49 outbreaks caused by influenza, the median attack rate in residents was 33% (range 4‐94%), and 23% (range 3‐58%) among staff, with a median case‐fatality rate for residents of 6.5% (range 0‐55%). Over three consecutive 9‐year time periods between 1980 and 2008, there was no observed decrease in attack rates or case‐fatality rates; nevertheless, these data should be interpreted cautiously as antiviral use and the stringency of application of infection prevention and control (IPC) practices has changed over time.

Exposure to influenza in residents of LTCFs for the elderly increases their risk of respiratory‐origin hospitalisation (relative risk [RR] 1.43 [95% CI 0.99‐2.08]), and particularly increases the risk of death due to a respiratory cause (RR 2.77 [95% CI 1.55 to 4.91]) compared to unexposed residents, despite high levels of vaccination among the residents (93%).^18^

Bronchitis and pneumonia, either primary influenza pneumonia or secondary bacterial pneumonia, are the most common respiratory complications of influenza infection, but infection may also cause extrapulmonary cardiovascular, neurological and musculoskeletal manifestations. In a retrospective cohort study of nursing home residents in 381 nursing homes across three seasons, estimated for the 63% of residents with comorbid conditions, influenza contributed to approximately 28 hospitalisations, 147 courses of antibiotics and 15 deaths per 1000 person‐years annually; a higher burden than residents without comorbid conditions but in whom there were still seven hospitalisations, 99 excess antibiotic prescriptions and six deaths per 1000 residents annually.^16^ Increased age itself is a recognised risk factor for serious influenza infection. A systematic review evaluating populations at risk of severe influenza‐related illness found that for seasonal influenza (H3N2 or type B), there was raised risk of hospitalisation (odds ratio [OR] 4.65, 95% confidence interval [CI] 1.74 to 12.41) and risk of death (OR 2.95, 95% CI 1.53 to 5.70) among elderly people (>65 years) compared with non‐elderly people.^19^ Modelling studies suggest that the burden on the health services is particularly onerous for those aged 75 and above, with an estimated 36% of all influenza‐attributable respiratory hospitalisations and 74% of all influenza‐associated deaths occurring in this age group in the UK over 13 seasons,^20^ and accounting for 52% of total hospital bed occupancy and 69% of excess bed days occupancy in England over a similar number of seasons.^21^

**Key Points 1**

- LTCFs are susceptible to seasonal influenza outbreaks, which may be explosive and with high attack rates in residents.
- Written IPC policies, vaccination policies for residents and staff, provision of ongoing staff IPC training and the facilities required to promote compliance with IPC practices should be in place throughout the year.
- Although influenza vaccine efficacy is lower in the elderly and in the presence of comorbidities compared to healthy younger adults, vaccination of LTCF residents remains a major public health tool and is recommended.
- Vaccination of LTCF staff is recommended and should be encouraged. Although evidence for a protective effect of HCW vaccination to protect the frail and elderly is weak, HCW vaccination can help to protect themselves, maintain the workforce during an outbreak and act as a barrier to transmission of infection to the vulnerable.

## 3 EPIDEMIOLOGY

Outbreaks of influenza (and other respiratory virus pathogens) in LTCFs in the Northern Hemisphere occur most commonly during the winter but may occur at any time of year, particularly in the autumn months, usually due to circulation of influenza A(H3N2) and before seasonal vaccination campaigns have been fully implemented or when matching is poor; also in the spring when influenza B often peaks^22^ and when antibody titres may have declined in the vaccinated.^23^, ^24^

Influenza virus replicates in the epithelium of the upper and lower respiratory tract, with infected hosts releasing virus into the environment during breathing, talking, coughing and sneezing, producing a spray of virus‐containing particles ranging in size from 0.01 to 500 μm.^25^ Transmission of influenza may occur by three routes: droplet (larger‐sized particles too large to be inhaled into the lungs and which settle quickly to the ground or other surface within 2 m of the source); aerosol or droplet nuclei (small particles <5 μm which can remain suspended in the air much longer and are potentially inhalable into the lower respiratory tract); and contact (transfer of infectious particles to the mucous membranes directly or indirectly through contaminated objects). The relative importance of each of these routes in influenza transmission is unclear, and the contribution of aerosolised infectious droplet nuclei has been particularly contentious.^26^, ^27^, ^28^, ^29^, ^30^, ^31^ However, most influenza transmission is short‐range, and when it has occurred over longer distances, contact transmission has generally not been ruled out.^32^ Transmission studies often do not control for confounders such as vaccination status, handwashing practice, supershedders, amount of coughing, ward layout, surface contamination and ventilation,^33^ and further studies which control for these are required in this area.

The incubation period of influenza is typically short, usually reported as ranging from 1 to 4 days,^34^ with a serial interval (time between symptom onset in a secondary case and that of its primary case) of 2.2 to 3.5 days for influenza A and 3.4 to 4.9 days for influenza B.^35^ The relatively short incubation period and serial interval enables the virus to spread rapidly through communities, so mitigation measures such as isolation and transmission‐based precautions should be instigated as soon as a case of suspected influenza is identified to minimise the risk of transmission to contacts.

Viral shedding has generally been considered to be a proxy for influenza infectiousness,^36^, ^37^, ^38^ peaking 1 to 3 days after symptom onset with most healthy volunteers clearing virus by day 6 to day 7.^34^ However, a recent study of household influenza transmission found at most only a weak association between viral load in nose and throat swabs and infectivity, possibly due to the weak correlation between virus concentration in exhaled breath and nose and throat samples, or due to the intensity of household transmission so that even those with low viral loads are still capable of infecting those around them.^39^ Pre‐symptomatic shedding may occur in up to one‐third of cases,^40^, ^41^, ^42^, ^43^ and prolonged viral shedding has been reported in children,^42^, ^44^, ^45^ in patients hospitalised with severe influenza^46^ and in immunocompromised patients,^47^ in whom prolonged shedding may last weeks or even months.^48^, ^49^ The transmission dynamics of influenza infections in residents of LTCFs have not been studied; age >65 years and the presence of major comorbidities were associated with prolonged shedding of virus and higher viral load in a prospective observational study of hospitalised influenza patients^46^; these findings may raise the possibility of prolonged shedding in LTCF residents.

Transmission of influenza from healthcare workers (HCWs) to hospital patients, including those in geriatric facilities, has been well documented using epidemiological linkage, nucleotide sequence analysis and contact tracking data^50^, ^51^, ^52^, ^53^ and case reports of outbreaks of influenza‐like illness in care facilities indicate that staff can transmit the virus to residents.^54^, ^55^ A systematic review comparing the incidence of influenza in HCWs with other workers not working in a healthcare setting and taking vaccination status into account, found estimated incidence rates (IRs) for all influenza infections (defined as a ≥4‐fold increase in antibody titre over the influenza season and including asymptomatic infections) of 18.7/100 population/season (95% CI 15.8 to 22.1) for unvaccinated HCWs and 6.5/100 population/season (95% CI 4.6 to 9.0) for vaccinated HCWs, both higher than the IRs in unvaccinated and vaccinated other workers (5.4/100 population/season [95% CI 3.0 to 9.8] and 1.2 [95% CI 0.9 to 1.7] respectively).^56^ However, no difference was observed between IRs for symptomatic infection confirmed serologically in HCWs compared to other workers; this overall lack of consistency in findings between overall and symptomatic infections indicates the need for cautious interpretation. The observed variability might be explained by HCWs being at higher risk of asymptomatic or subclinical infection, indicating that HCWs may act as an infective pool to transmit influenza to frail elderly people. Furthermore, a study of HCWs in an acute hospital during a mild epidemic season, found that 23% had serological evidence of new influenza infection during the season, implying a potential transmission risk to patients as between 28% and 59% of infected workers had subclinical infections and continued to work.^57^ Although the role of asymptomatic people and those with only mild symptoms in spreading influenza is uncertain, HCWs often continue to work despite having symptoms and may act as a source of infection to those in their care.^58^, ^59^, ^60^ Nursing home aides in particular have been shown in one Swedish study to be the occupational group at significantly greatest risk of continuing to work despite the feeling that, in the light of their perceived state of health, they should have taken sick leave.^61^ However, in reality the employment status of many LTCF staff is often precarious and taking unpaid sick leave may result in adverse economic consequences.

## 4 ROUTINE AND PRE‐OUTBREAK PREVENTION MEASURES

### 4.1 Planning, training and education

LTCFs have a broad staff base and may include people with little or no formal healthcare training. Depending upon the type of facility and the nursing needs of the residents, services are provided by a range of staff including care assistants with few formal healthcare qualifications, registered nurses, domestic staff, catering and administrative staff, with additional ambulatory health services usually provided by external contractors such as general practitioners (GPs) and other healthcare professionals not directly affiliated to the facility. Managers of LTCFs have an important role in ensuring that all staff have ongoing training on the importance and practice of IPC, and that the facilities are available for IPC measures to be implemented to a satisfactory level and with standard precautions being used at all times, regardless of the detection of a suspected outbreak. Written policies should be in place in every LTCF outlining: resident and staff influenza immunisation policy; a written outbreak management plan which includes outbreak recognition (definitions, thresholds for suspicion of an outbreak), identification of communication channels, operational measures including active surveillance, staff contingency plans, visitor restriction policies, and consideration of antiviral treatment and prophylaxis strategy; a policy for ill staff to remain off work; a policy for dealing with visitors with symptoms of an acute respiratory tract infection.

During the Severe Acute Respiratory Syndrome outbreak, compliance with IPC measures was found to be associated with HCWs’ perception that the facilities in which they worked had clear IPC policies and protocols, and that the management had a positive attitude towards occupational health and safety and provided training in IPC practices.^62^ Managers in LTCFs therefore have a pivotal role in creating a strong institutional climate in which staff feel valued, with continuous accessibility to the training resources, clear IPC policies and supplies and facilities required to promote compliance with IPC practices.

### 4.2 Vaccination of LTCF residents

A WHO strategy and action plan for healthy ageing in Europe 2012‐2020 recognised the benefit of proper vaccination strategies against infectious diseases, including influenza, both in older people and for health and social care workers in contact with them, and proposed priority interventions including national immunisation schedules and the implementation of infectious disease control programmes in institutions.^63^ Furthermore, a WHO position paper published in 2012 recommended that elderly persons ≥65 years and people with specific chronic diseases should be considered for influenza vaccination.^64^ Vaccination coverage of the elderly varies considerably between European countries with recent uptake rates reported between 1% and 77.4% and with only two countries (the Netherlands and the UK) achieving, or almost achieving, the WHO target of 75% coverage in the elderly.^65^ For residents of LTCFs, recent data available from only three countries indicated vaccination coverage rates of 71% to 89%.^65^

Vaccination of residents remains an important public health tool to protect the elderly and those with underlying conditions, but there is uncertainty about how effective immunisation is at an individual level in LTCF residents. A systematic review assessing the effectiveness of influenza vaccine in people 65 years or over, with separate analyses for those living in nursing homes and community dwelling older people,^66^ concluded that for elderly people living in closed communities vaccination may be slightly to moderately more effective than no vaccination at preventing influenza‐like illness (ILI) (24% [95% CI 12‐34%]), pneumonia (47% [95% CI 34‐57%]), hospitalisation (49% [19‐68%]), overall mortality (60% [95% CI 23‐79%]) and mortality from influenza or pneumonia (54% [95% CI 37‐67%]), although no significant protective effect against proven influenza was found. A later systematic review^67^ also found that vaccination may have a small significant protective effect against pneumonia (37% [95% CI 18‐53%]) and mortality from influenza and pneumonia (34% [95% CI 10‐53%]), in institutionalised older adults ≥60 years; also a trend towards protection against ILI (21% [95% CI −3 to 39%]). The authors did not address the effectiveness of vaccination against all‐cause mortality and, due to an insufficient number of studies, were unable to perform meta‐analyses for laboratory‐confirmed influenza or hospitalisation. The quality of the evidence is very weak in both reviews and does not definitively answer the uncertainty regarding the effectiveness of influenza vaccination in older people living in LTCFs. In particular, selection bias may occur by targeting the frail for immunisation; conversely people who are particularly frail or close to death may not receive vaccine resulting in overestimation of the effectiveness of vaccine on mortality (healthy recipient effect).^68^, ^69^ Although there is some indirect evidence of influenza vaccine effectiveness (IVE) against hospitalisation, pneumonia and death from studies which have controlled for multiple confounders and compared summer and winter mortality in vaccinated and unvaccinated elderly people,^70^, ^71^ accurate proof of IVE requires adequately powered studies using laboratory‐confirmed outcomes, in which confounders are controlled for and which comprehensively monitor morbidity and mortality.^72^

### 4.3 Vaccination of LTCF staff

Infection in HCWs affects not only themselves and their immediate family but may further inhibit efforts to control an outbreak if staff shortages result in remaining staff having to care for both affected and unaffected residents.^73^ On this basis HCWs are recognised as a priority group for vaccination and are generally recommended to receive it.^64^, ^74^

High rates of staff vaccination in LTCFs have been demonstrated in several studies to decrease the risk of all‐cause mortality and ILI in frail elderly residents, with lowest rates when both HCWs and patients had high vaccine coverage rates.^75^, ^76^, ^77^, ^78^, ^79^ This has been somewhat refuted by a systematic review of four cluster‐randomised controlled trials (RCTs) and one cohort study which suggested that offering vaccination to HCW caring for people aged over 60 in LTCFs may have little or no effect on laboratory‐confirmed influenza (LCI) (Risk difference (RD) 0 (95% CI −0.03 to 0.03) and respiratory‐related hospitalisation in residents (RD 0 (95% CI −0.02 to 0.02), although there may be a small decrease in lower respiratory infections from 6% to 4% in homes where HCW vaccination is offered (RD −0.02 [95% CI −0.04 to 0.01]).^80^ The effects on deaths due to LRTI and all‐cause mortality were not evaluated by meta‐analysis, but reductions in all‐cause mortality were noted in the individual studies (ORs ranging from 0.56 [95% CI 0.4‐0.8] to 0.80 [95% CI 0.67 to 0.97]).^75^, ^76^, ^77^, ^79^ The authors called for high quality RCTs to address methodological flaws they identified in the included studies, and to test the effectiveness of co‐interventions such as handwashing and face‐mask use in combination with HCW vaccination. Another systematic review^81^ found a significant reduction in ILI and all‐cause mortality in residents associated with vaccination of HCWs (42% and 29% reductions respectively), but no significant reductions for laboratory‐confirmed influenza or all‐cause hospitalisations. Overall the authors rated the quality of evidence as very low to moderate for the different outcomes, and concluded that HCW influenza vaccination can enhance patient safety. Protective effects of HCW vaccination against non‐specific outcomes may be an indication of unaccounted cluster biases, such as differences in handwashing or other IPC precautions and warrants further investigation.^82^ Evidence from observational and modelling studies suggests a likely proportionate effect of HCW vaccination coverage on patient protection, although no clear threshold for HCW vaccine uptake above which the protective effect on residents increases substantially has been established.^83^, ^84^, ^85^, ^86^, ^87^

Inactivated trivalent vaccines have been found to have a protective effect against proven influenza in healthy adults (overall protective effect in vaccine‐matched and poorly matched seasons 62% [95% CI 56‐67%]).^88^ A systematic review specifically addressing the effectiveness of seasonal influenza vaccination in HCWs found just one study reporting laboratory‐confirmed influenza in this group with a reported IVE of 88% (95% CI 59% to 96%, *P*=.0005).^89^

Although the currently available evidence may be weak for HCW vaccination to protect the frail and elderly, there is also generally no evidence against it. Therefore, it remains a biologically plausible intervention to provide individual protection to the HCW, act as a barrier against spread of infection and to help reduce the risk associated with influenza infection and prevent staff absenteeism. However, poor vaccine uptake by HCWs has been well documented. In Europe, coverage of HCWs (including those working in LTCFs) varies between countries and is generally much lower than for other vaccination targeted groups, ranging from 9.5% to 75% with a median vaccination coverage rate of 28.6%.^65^ In the United States, vaccination rates of 50‐70% have been reported for LTCF workers,^78^, ^90^, ^91^ with coverage consistently lower than among staff working in hospital settings.^91^ Reasons given for declining vaccination include fear of side effects, lack of concern or perception of risk, doubts about vaccine efficacy, lack of availability or inconvenient delivery of vaccine, avoidance of medications and dislike of injections.^92^ Although mandatory vaccination is effective if it can be implemented,^93^ it is not legally enforceable in all countries and settings, and infection rates after the implementation of mandatory vaccination have not been studied. The United States Department of Health and Human Services advocates a 90% HCW vaccination rate by 2020 but as yet there have been no RCTs of transmission with very high HCW vaccination rates.^33^ Data suggest that other interventions such as easier access to vaccine, educational activities, reminders and organisational changes can increase uptake in proportion to the number of interventional components, although most studies have only evaluated their effect on a short‐term basis whereas long‐term intervention programmes will be necessary to demonstrate a sustainable effect on uptake.^94^ To address low uptake of seasonal influenza vaccination in priority target groups, a project called TIP FLU was initiated in 2013 by the WHO Influenza and other Respiratory Pathogens programme. Adapted from the Tailoring Immunization Programmes (TIP) approach, it is based on social and behavioural change models and provides tools to identify priority populations, determine barriers and enablers to vaccination, and to implement evidence‐based interventions. A guide and case study of the application of TIP FLU in Montenegro have been published to assist policymakers and programme managers to increase vaccine uptake among HCWs.^95^, ^96^

## 5 EARLY RECOGNITION OF INFLUENZA IN LTCFS

### 5.1 Diagnosis in residents

Early recognition of influenza in residents of LTCFs may be problematic due to non‐specific symptoms and the possibility of atypical presentation and lack of fever in the elderly with influenza.^97^, ^98^ Influenza may present as sudden, unexplained deterioration in physical or mental ability or exacerbation of an underlying condition with no other known cause.^15^ The use of surveillance case definitions for ILI in these populations may therefore miss cases, especially if they present without fever. Furthermore, other underlying conditions may impair residents’ abilities to verbalise their symptoms. This may impede the early implementation of control and treatment strategies.^99^ The precise definition of ILI may vary from country to country; the WHO global surveillance case definition of ILI is an acute respiratory infection with measured fever ≥38°C and cough and onset within the last 10 days,^100^ whereas that of the Centers for Disease Control and Prevention (CDC) and European Centre for Disease Prevention and Control (ECDC) definition is sudden onset of symptoms and at least one of four systematic symptoms (fever or feverishness, malaise, headache, myalgia) and at least one of three respiratory symptoms (cough, sore throat, shortness of breath).^3^, ^101^

To confirm an outbreak, reverse transcriptase polymerase chain reaction or viral culture are the preferred methods of laboratory testing. Rapid point‐of‐care diagnostic tests can produce a result within 30 minutes but have lower sensitivity (median 70‐75%)^102^ and there may be variability between different age groups and influenza subtypes,^103^ although they may still be useful in outbreak situations, for example for rapid identification of influenza infection where timely access to more sensitive laboratory testing is unavailable or delayed. However, clinical judgement is required to interpret negative rapid test results for individual patients during an outbreak and negative rapid test results may not justify delaying the instigation of outbreak control measures if there is clinical and epidemiological suspicion.

## 6 OUTBREAK CONTROL MEASURES

Outbreak definitions vary between countries and are frequently based on the number of cases in a unit during a specified period of time. Although influenza may cause sporadic infection in LTCFs, given the vulnerability of the population and the propensity to spread rapidly, it is wise to have a low threshold for declaring an outbreak and commencing control measures, ideally before virological confirmation. One case may be indicative of incubating infection in exposed persons, so these should be actively sought through daily surveillance of temperature and symptoms in all residents and staff.

Infection prevention and control strategies in healthcare facilities are commonly based on early recognition and controlling the source of the pathogen, administrative controls, environmental hygiene and engineering controls, and the use of personal protective equipment (PPE). In addition to standard IPC precautions, which are routine measures that should be practised at all times by all staff and with all residents,^104^ contact and droplet transmission‐based precautions should be implemented as required when residents are suspected to be infected.

The use of hygiene measures, such as hand hygiene, respiratory etiquette and appropriate use of PPE may reduce the transmission of respiratory viruses^105^, ^106^, ^107^ and are central to IPC programmes in LTCFs. Compliance with these measures may be an issue and there is currently a paucity of directly observed studies of handwashing and mask‐wearing.^33^ There is some evidence that the wearing of a surgical face mask by an infected person decreases their infectiousness to others^108^, ^109^ and may be considered for infected LTCF residents, particularly if they have to be moved outside their own room or cohort area; although in reality this measure may not be tolerated by some individuals. Residents sharing a room with an influenza‐infected roommate have three times the risk of acquiring infection than those in single rooms.^110^ Although there is little convincing evidence that social distancing and isolation are effective in reducing transmission,^105^, ^107^ isolation of infected residents in single‐occupancy rooms and limitation of social activities minimises transmission opportunities, although this may not be practical in many LTCFs with limited accommodation types, in which case cohorting infected residents together (with separate cohorts of confirmed cases and those with the same suspected diagnosis on the basis of epidemiological and clinical information if possible) is an alternative to isolation. A balance may need striking between the strict enforcement of social isolation and visitor restrictions and psychological welfare in vulnerable populations. Other measures to control transmission will include closure of the facility to new admissions based on risk assessment, limitation of visitors, cohorting staff to avoid crossover of care for infected and asymptomatic residents, excluding staff with ILI symptoms, rostering vaccinated staff to care for infected residents, and preventing unvaccinated staff from working in other healthcare facilities during the outbreak. As influenza virus has been shown to survive on hands and inanimate surfaces from a few hours for up to several days,^111^ regular and thorough hand hygiene and enhanced environmental cleaning may reduce contact transmission, and there is evidence that even simple, readily available, easy to handle products such as 1% bleach and detergents like 0.01% washing‐up liquid are effective in killing influenza virus depending upon the material and situation and can be used even in low‐resource settings.^112^

### 6.1 Antiviral treatment

Early recognition of an influenza outbreak in a LTCF can facilitate timely antiviral treatment and prophylaxis to end the outbreak and thus avoid influenza‐related complications in exposed residents. The neuraminidase inhibitors (NAIs) oseltamivir and zanamivir are currently authorised for the treatment and prophylaxis of influenza in Europe and the United States. However, their effectiveness has been subject to much debate^113^ and many clinicians have felt confused about when to use them appropriately.

A modest but significant reduction in time to first alleviation of symptoms has been consistently shown in meta‐analyses of RCTs of previously healthy people, representing a 10 to 15% reduction in overall duration of symptoms in those treated with an NAI compared with those receiving placebo,^114^, ^115^, ^116^ with similar reductions noted from analysis of observational studies.^117^ This effect appears to be somewhat attenuated in the elderly.^115^, ^118^ In general there is a lack of credible evidence from RCTs that NAIs reduce the risk of hospitalisation and pneumonia,^114^, ^115^, ^118^ although a significant reduction was seen in hospitalisation in the influenza‐confirmed intention‐to‐treat population in one analysis (RR 0.37 [95% CI 0.17 to 0.81]),^115^ and reduced rates of lower respiratory tract complications were seen in two meta‐analyses (RR 0.56 [95% CI 0.42 to 0.75]^115^ and 0.55 [95% CI 0.33 to 0.90]).^114^ However, RCTs are generally underpowered to evaluate the effects of treatment on complications due to the rarity of such events, and lack of precise outcome definition in many trials makes comparison of findings difficult. Meta‐analysis of observational studies indicated a potential effect of oseltamivir in reducing hospitalisation in the general population (all ages) compared with no antiviral (OR 0.75 [95% CI 0.66 to 0.89]), but no significant effect was found for inhaled zanamivir (OR 0.66 [95% CI 0.37 to 1.18]).^117^ Individual patient data meta‐analysis from 29 234 people of all ages hospitalised with 2009 H1N1 pandemic influenza showed that deaths were reduced when treated with NAIs within 48 hours of onset (OR 0.81 [95% CI 0.70 to 0.93]), supporting the use of early NAI treatment in those who require hospitalisation.^119^ A reduction in mortality risk for patients receiving oseltamivir compared to those not given antivirals was also noted in a meta‐analysis of three observational studies (OR 0.23 [95% CI 0.13 to 0.43]) but the quality of evidence was low, and related only to oseltamivir.^117^

Uncertainties remain about the effectiveness of treatment in high‐risk populations such as LTCF residents as much of the available evidence relates to previously healthy younger adults. However, given the high risk of severe complications and deaths among the elderly from influenza virus infection, where influenza antivirals are available, treatment of symptomatic LTCF residents is generally recommended to be started immediately.^3^, ^120^

### 6.2 Antiviral prophylaxis

Prophylaxis with antivirals is intended to prevent transmission of influenza virus to people who are not exhibiting ILI but who have or may have been exposed. There is a paucity of evidence from recent studies to inform a single approach for antiviral prophylaxis use in LTCFs, so decisions should be based on clinical judgement and outbreak severity.^121^ Prophylaxis with oseltamivir or zanamivir was shown to be more effective than placebo at preventing symptomatic influenza in individuals in the community (oseltamivir RR 0.45 [95% CI 0.30 to 0.67]; zanamivir RR 0.39 [95% CI 0.22 to 0.70]) and in household contacts (oseltamivir RR 0.20 [95% CI 0.09 to 0.44]; zanamivir RR 0.33 [95% CI 0.18 to 0.58]) in a recent meta‐analysis of RCTs.^114^ A reduced risk of LCI with oseltamivir and zanamivir prophylaxis was found in a further systematic review which also included data from observational studies, for both individuals (oseltamivir OR 0.11 [95% CI 0.06 to 0.20]; zanamivir OR 0.23 [95% CI 0.16 to 0.35]) and households (oseltamivir OR 0.23 [95% CI 0.09 to 0.59]; zanamivir OR 0.18 [95% CI 0.10 to 0.31]).^122^ However, direct evidence of effectiveness in reducing symptomatic influenza in the frail elderly living in institutions is sparse; a non‐significant protective trend with post‐exposure zanamivir prophylaxis has been shown in one study (RR 0.08 [95% CI 0.01 to 0.63])^123^ but data for the effectiveness of post‐exposure oseltamivir in this setting are lacking.^124^ Other studies have seen a non‐significant effect of post‐exposure prophylaxis and seasonal prophylaxis with zanamivir.^124^, ^125^

Prophylaxis for all residents in LTCFs experiencing an outbreak, regardless of vaccination status is recommended by the US Centers for Disease Control and Prevention (CDC)^3^; the European Centre for Disease Prevention and Control (ECDC) expert opinion on the use of antivirals for prophylaxis recommends consideration of antiviral prophylaxis for residents of LTCFs, especially for those who are unvaccinated or immunocompromised who do not respond to vaccination.^126^ This may be particularly important during years when IVE is expected to be low due to vaccine strain mismatch. But the relatively low effectiveness of influenza vaccine in the elderly population even in well‐matched years should also be taken into consideration.

There is a lack of studies evaluating the effectiveness of giving prophylaxis to HCWs in LTCFs. The CDC recommends consideration of prophylaxis for unvaccinated HCWs caring for people at high risk of complications, and for all employees regardless of vaccination status if an outbreak is due to a strain which is poorly matched to the current vaccine strains.^3^ To protect vulnerable people, ECDC also recommends consideration of prophylaxis for HCWs, particularly when low IVE is expected due to strain mismatch.^120^ As IVE is lower in those who are elderly and frail than in younger healthy people, it would seem prudent to have a low threshold for offering prophylaxis to those caring for them, and particularly so if chains of transmission from a resident to staff are described; or from a resident to staff to resident.

## 7 CONCLUSION

Seasonal influenza infection outbreaks are a significant problem in LTCFs both in terms of morbidity and mortality for individuals as well as putting additional strain on already overburdened health services. With an increasingly elderly population, the demand for LTCFs is likely increase over the coming years. Many basic and clinical questions remain unanswered about the transmission, prevention and treatment of influenza in these institutions and further evidence is needed to determine which interventions, or combination of interventions and hygiene practices are most efficacious in these settings. Although vaccination for residents and staff forms the cornerstone of preventive influenza policy in LTCFs, and vaccine coverage is high among residents in some countries, currently available vaccines are less effective in older people and those with comorbidities. Vaccination coverage among HCWs caring for residents of LTCFs is generally much lower than for those in other priority groups and efforts are required to improve this to protect the vulnerable, the individual HCW and the workforce. Although further studies of the efficacy of antiviral treatment and prophylaxis in LTCF setting are required, available data suggest that antivirals should be used early during the course of infection for treatment of residents and considered for prophylaxis during suspected or confirmed influenza outbreaks to reduce secondary transmission. The prevention and control of influenza in LTCFs requires a multifaceted approach; vaccination and antiviral policies form an important part of this, but strong managerial leadership, outbreak planning, and a well‐trained, educated and engaged workforce are pivotal to the successful implementation of IPC policies.

## CONFLICT OF INTERESTS

J.S.N‐V‐T. reports grants from World Health Organization, during the conduct of the study; and grants from F. Hoffman‐La Roche and non‐financial support from European Scientific Working Group on Influenza (ESWI), outside the submitted work. J.S.N‐V‐T and L.L declare financial support outside the submitted work from GlaxoSmithKline Biologicals SA for a systematic review of pandemic influenza vaccines which ended in 2016. J.S.N‐V‐T. is also Editor in Chief of Influenza and Other Respiratory Viruses but has played no role in the handling, peer‐review process or decision‐making regarding this manuscript. All of these functions were undertaken or organised independently by Dr. J. Wood, Senior Editor (Reviews). CSB declares no conflict of interests.

**Key Points 2**

- Early recognition of a potential outbreak is important to facilitate timely antiviral treatment, prophylaxis and instigation of IPC measures (prior to virological confirmation if appropriate).
- Rapid point‐of‐care tests may be useful, but negative tests do not exclude a diagnosis of influenza.
- Antiviral treatment of symptomatic residents is recommended to be started immediately given their high risk of complications.
- For asymptomatic residents, the decision to give antiviral prophylaxis should be made on an individual basis using clinical judgement and risk of exposure, but as influenza vaccine is considered less effective in the elderly, the threshold for offering antiviral prophylaxis to all residents should be low.
